# Internet Gaming Disorder Does Not Predict Mood, Anxiety or Substance Use Disorders in University Students: A One-Year Follow-Up Study

**DOI:** 10.3390/ijerph20032063

**Published:** 2023-01-23

**Authors:** Guilherme Borges, Corina Benjet, Ricardo Orozco, Yesica Albor, Eunice V. Contreras, Iris R. Monroy-Velasco, Praxedis C. Hernández-Uribe, Patricia M. Báez-Mansur, María A. Covarrubias Diaz Couder, Guillermo E. Quevedo-Chávez, Raúl A. Gutierrez-García, Nydia Machado

**Affiliations:** 1 Center for Global Mental Health, National Institute of Psychiatry Ramón de la Fuente Muñiz, Mexico City 14370, Mexico; 2 Facultad de Ciencias Administrativas y Sociales, Universidad Autónoma de Baja California, Ensenada 22860, Mexico; 3 Facultad de Psicología, Universidad Autónoma de Coahuila, Saltillo 25125, Mexico; 4 Secretaría de la Unidad Cuajimalpa, Universidad Autónoma Metropolitana, Mexico City 14387, Mexico; 5 Universidad La Salle Victoria, Ciudad Victoria, Mexico; 6 Coordinación de Investigación, Universidad la Salle Noroeste, Ciudad Obregón 85019, Mexico; 7 Coordinación de Psicología, Universidad la Salle Cancún, Cancún 77560, Mexico; 8 Facultad de Ciencias Sociales y Humanidades, Universidad De La Salle Bajío, Salamanca 36700, Mexico; 9 Instituto Tecnológico de Sonora, Ciudad Obregón 85059, Mexico

**Keywords:** internet gaming disorder (IGD), anxiety, depression, substance use disorders, longitudinal, epidemiology, Mexico

## Abstract

We seek to evaluate whether Internet Gaming Disorder (IGD) among university students in Mexico during their first year at university predicts a long list of mental disorders a year later, controlling for baseline mental health disorders as well as demographics. This is a prospective cohort study with a one-year follow-up period conducted during the 2018–2019 academic year and followed up during the 2019–2020 academic year at six Mexican universities. Participants were first-year university students (n = 1741) who reported symptoms compatible with an IGD diagnosis at entry (baseline). Outcomes are seven mental disorders (mania, hypomania, and major depressive episodes; generalized anxiety disorder and panic disorder; alcohol use disorder and drug use disorder), and three groups of mental disorders (mood, anxiety, and substance use disorders) at the end of the one-year follow-up. Fully adjusted models, that included baseline controls for groups of mental disorders, rendered all associations null. The association between baseline IGD and all disorders and groups of disorders at follow-up was close to one, suggesting a lack of longitudinal impact of IGD on mental disorders. Conflicting results from available longitudinal studies on the role of IGD in the development of mental disorders warrant further research.

## 1. Introduction

Since its inclusion in the Diagnostic and Statistical Manual of Mental Disorders Fifth Edition [1], Internet Gaming Disorder (IGD) has been associated with several mental disorders, particularly major depressive disorder, attention-deficit/hyperactivity disorder (ADHD), and obsessive-compulsive disorder [2]. In the DSM-5, IGD was given an initial provisional description; nine clinical symptoms were identified, and a suggested cut-off point provided (IGD was defined as presenting five or more out of the nine proposed symptoms; mild, moderate, and severe categories were named but not defined). Furthermore, an international consensus, led by Nancy Petry et al. (2014) [3], offered a more detailed description of the nine symptoms put forward by the DSM-5 and formulated the key elements for epidemiological studies on the issue. Since the publication of DSM-5 in 2013, research in this area has tended to be more homogenous in defining gaming using the nine symptoms suggested by the DSM-5. These nine symptoms are as follows: a preoccupation with/too much time spent gaming; a withdrawal feeling (being restless, irritated, angry, anxious, or sad) when gaming is removed; tolerance, i.e., needing to spend increasingly more time for excitement; unsuccessful attempts to control the amount of time spent gaming; loss of other interests; continued excessive use despite psychosocial problems; deceiving others regarding online gaming; gaming to escape one’s troubles and negative consequences.

Nevertheless, many of the previous reports in this area are drawn from cross-sectional studies in which it is impossible to separate cause-effect relations between IGD and mental disorders [4,5]. Moreover, much less information is available from follow-up studies, most of which focus on children and adolescents [6,7]. Young adults entering college are a population at risk of IGD because they spend large amounts of time using computers for learning, peer communication, and leisure [8]. Because those starting college also face stress from a new, demanding learning environment, they are also at risk of mental disorders [9]. Few epidemiological studies are available for IGD among college students, and only a handful are follow-up studies that would enable one to determine the role of IGD in the onset of mental disorders [10].

Results of previous studies on the causality between IGD mental disorders are inconsistent [11]. Some do not focus on IGD but on prior concepts such as problem gaming, and there is a lack of prospective data on the impact of IGD on mental health disorders in university students. The aim of this prospective cohort study of college students in Mexico was therefore to evaluate whether IGD in the first year of university predicts a long list of mental disorders a year later, controlling for baseline mental health disorders as well as demographics.

## 2. Materials and Methods

### 2.1. Study Design

This is a prospective cohort study with a one-year follow-up [12]. Participants were first-year university students who reported symptoms compatible with a diagnosis of IGD at entry (baseline). Outcomes include seven mental disorders and three groups of mental disorders (see below for list) at the end of the one-year follow-up. We used a census sampling scheme to recruit as many schools, and students within those schools, as possible.

### 2.2. Sample and Procedure

Participants were first-year university students from one cohort of PUERTAS (University Project for Healthy Students) [13], a web-based survey conducted during the 2018–2019 academic year and followed up during the 2019–2020 academic year at six Mexican universities (two public and four private) as part of the World Health Organization World Mental Health International College Student initiative (WMH-ICS) [14]. These students were the first cohort to be given an IGD scale [15].

All first-year students aged 18 or older were eligible; they were recruited through events they attended and given a general link to the online survey (new student orientation and first-year courses). Twelve months later, all students who had completed a survey in their first year were sent a personalized email link to answer the follow-up survey. In total, 8122 first-year students participated in the baseline survey, with 1741 taking part in the follow-up. Participation was confidential and voluntary, and required informed consent. The Research Ethics Committee of the National Institute of Psychiatry in Mexico approved the study (CEI/C/032/2016). The overall baseline response rate for all universities in the broader PUERTAS study was 79.3%.

### 2.3. Measures

The online, self-report survey was developed for the WMH-ICS Initiative [14] and comprises the validated measures described below.

#### 2.3.1. Baseline Control Variables

These were socio-demographic variables and consisted of sex (“female”, “not female”), and age. Fully adjusted models also included three groups of mental disorders at baseline: mood, anxiety, and substance use disorders (see below for list).

#### 2.3.2. Exposure Variable: Internet Gaming Disorder at Baseline

At baseline, all participants were asked whether they had played video games in the past twelve months. Video game users (hereafter called “gamers”) are those who reported having played video games (on a computer, smartphone, console, or any other electronic device) in the past. To avoid unnecessary respondent burden, the full IGD scale was only administered to gamers who had screened positive for the following: if they played, on average, at least one day a week, and the duration of their gaming (on weekdays or at the weekend) was at least thirty minutes (hereafter called “active gamers”). The section on IGD consisted of twenty-three items based on the nine symptoms or domains described in the DSM-5 and formulated by an international consensus led by Nancy Petry, which included an English version and a Spanish translation [15,16]. As per DSM-5, the presence of five out of nine symptoms means that someone has probable IGD. A description of the individual symptoms of IGD, together with the psychometric properties of the scale, was reported elsewhere.

The IGD scale showed unidimensionality and had factor loadings of between 0.694 and 0.838, and a Cronbach’s α = 0.816. The four symptoms derived from gaming, combined on a continuum with the five symptoms of substance disorders, suggest an appropriate representation of the psychological problems some gamers may present.

#### 2.3.3. Outcome Variables at One-Year Follow-Up: Mental Disorders

The Composite International Diagnostic Interview Screening Scales (CIDI-SC) [17] assessed the DSM diagnostic criteria for twelve-month symptoms of mood disorders: mania, hypomania, and major depressive episode; anxiety disorders: generalized anxiety disorder and panic disorder; and substance use disorders: alcohol use disorder and drug use disorder. These are called screening scales because they are non-clinician diagnosed, self-administered, and self-reported measures. However, they do assess each of the diagnostic criteria for these disorders. The CIDI-SC scales are consistent with the blinded clinical diagnoses in the AUC = 0.70–0.78 [17] range. The AUDIT screening scale [18] assessed alcohol disorder and is consistent with clinical diagnoses in the AUC = 0.78–0.91 [19] range. Although these self-report scales cannot be considered a clinical diagnosis of these disorders, they screen for clinically significant symptoms.

### 2.4. Analytic Approach

We calculated the rates for each of the seven outcomes through the weighted proportion of those with each outcome at follow-up among those with and without IGD at baseline. Log-binomial models examined the strength of individual-level associations (prevalence rate ratios [PR] in the bivariate model and risk ratios [RR] in the fully adjusted models) between the baseline predictor variables and the occurrence of our outcomes at follow-up [20,21]. We constructed two series of models. A first series predicted the likelihood of each outcome at follow-up, among students with or without IGD at baseline, a crude estimate of PR. A second series of models predicted the same outcomes at follow-up, adjusted for demographics and any mood disorder, anxiety disorder, or substance use disorder at baseline. These final models, as specified by VanderWeele et al. [22], are designed for longitudinal data, controlling for baseline confounders, outcome, and exposure, and can be interpreted as measures of causal links between exposure at baseline (IGD) and outcomes at follow-up (RR). We therefore reported these exponentiated coefficients as Risk Ratios, since controlling for prior values of the outcome enables us to rule out reverse causation more confidently.

All data analyses were adjusted for nonresponse weights, to offset potential non-response bias at follow-up, by sex and age [23] using Stata 17 [24].

## 3. Results

A total of 11,099 first-year students from participating universities enrolled in 2018, 8122 of whom participated in the baseline survey (with 8045 completing the IGD section). Of these, 1741 students participated in the one-year follow-up survey (equivalent to a participation rate of 21.4%), and 1731 had valid information for all the main variables in our analysis. Since those who completed the follow-up (n = 1741) were less likely to be male, or older (age 20+), than those who did not participate (n = 6381), all data analysis was weighted by sex and age to match the follow-up participants with the full baseline sample, as described in the Methods section. Further analyses of potential nonresponse bias showed no such bias in eight of our nine mental disorder outcomes; differences were only observed in major depression in baseline prevalence among those who participated in the follow-up compared to those who did not (*p* < 0.001).

Table 1 presents the distribution of our outcomes at follow-up, broken down by IGD at baseline (our exposure variable, prevalence 6.6%). Except for hypomania (1.1% among those without IGD and 0.7% among those with IGD) and panic disorder (5.7% among those without IGD and 3.9% among those with IGD), all the outcomes at follow-up were more frequently reported by those with IGD at baseline than those without.

Table 2 shows the results of the crude and adjusted models. Of the crude models, IGD at baseline was only associated with mania at follow-up, with a 3.51-fold increase in the likelihood of a mania episode. With two exceptions, all the disorders showed increased but non-significant prevalence ratios, and hypomania and panic disorder alone showed a decreased PR, which was also non-significant. Fully adjusted models, that included baseline controls for groups of disorders, rendered all associations null. The associations between baseline IGD and all disorders and groups of disorders was close to one, suggesting a lack of longitudinal impact of IGD on mental disorders.

## 4. Discussion

These findings contribute prospective evidence on the impact of IGD on other mental health problems, finding null associations. In short, our results suggest that a baseline IGD is not longitudinally associated with seven mental disorders in a one-year follow-up of first-year college students at a handful of universities in Mexico. These results contrast with those of another longitudinal study of university students in China, in which the authors reported that “internet addiction” at baseline had a predictive effect on follow-up depression, their only mental disorder outcome [10]. Another study in China reported that “problematic gaming” impacted both depression and symptoms in a nine-month follow-up of university students [25]. Our results are nevertheless similar to a survey in Norway, albeit of a younger population, which found that IGD was not longitudinally associated with depression, anxiety, ADHD, or a combination of oppositional defiant disorder (ODD) and conduct disorder (CD) [11]. Another study in a population of adolescents also found “pathological gaming” to increase depression, anxiety, and social phobia [26], while Wartberg found IGD to be prospectively associated among adolescents only with “emotional distress”, but not antisocial behavior, anger control, or hyperactivity/inattention [27]. The lack of associations between IGD and alcohol or drug use disorders in our study is similar to the results of research on adolescents in Norway, which found the amount of gaming to be associated with longitudinally measured depression, conduct problems, and aggression, but not heavy episodic drinking [28].

It is unclear whether the results of studies of IGD and mental disorders among children and adolescents can be extrapolated to young adults, a group that has been much less surveyed as regards these issues. Overall, the conflicting results of the current literature on whether IGD, and other related types of internet addiction, increases the occurrence of common mental disorders are a reflection of what a group of researchers in this area have identified as the lack of robust methodologies. They argue that research on the issue suffers from, “samples [that] are often quite small…The lack of sound assessment tools—particularly the lack of agreed-upon diagnostic criteria and standardized diagnostic interviews…”, and systematic bias from, “the use of convenience samples, lack of pro-active recruitment, inadequate assessment of confounding variables, and a dearth of representative and longitudinal studies” [29].

Our survey has significant limitations to consider. We only evaluated the impact of IGD at one year, rather than at the end of every year of college. Persistent IGD may have a cumulative negative impact over the years, whereas those with transitory or remitted IGD may have a more limited impact. This study has only one follow-up and some of our control variables may have changed during the short follow-up period. Longer follow-up studies, with more intermediate data points, are required. Because the response rate at follow-up was low, we weighted the data to adjust for non-response bias in sex and age. However, the sample may be biased in other ways. Finally, the universities included were both public and private, but were not randomly selected, meaning that study participants are not representative of all university students in Mexico.

Despite its limitations, this study provides evidence for the lack of effect of IGD upon a long list of common mental disorders. Because our students are not a random sample of unknown gamers from internet sites of video gamers, we have information on the basic demographic distribution and control of a series of key mental health disorders that may confound the relationship between IGD and mental health. By using a longitudinal approach, we provided stronger evidence for directionality and causality than correlational studies, and our controls for all mental health symptoms at baseline allowed us to rule out the fact that the lack of association between these symptoms and IGD could be explained by unmeasured, confounding mental health issues. Conflicting results for available longitudinal studies on the role of IGD in mental disorders warrants further research.

## 5. Conclusions

This longitudinal analysis suggests a lack of association between the alleged impact of IGD and mental disorders. It contributes to the scant literature on IGD among young adults based on prospective evidence, going further than cross-sectional studies on the issue of the short-term (one year) impact of IGD on other mental health problems, finding null associations. While more work is needed in this area, it is clear that the large comorbidity results reported from cross-sectional surveys between IGD and other mental disorders are not replicated longitudinally.

## Figures and Tables

**Table 1:** Distribution of individual and groups of disorders at follow-up, by baseline Internet Gaming Disorder (n = 1731).

	DSM-5 IGD (Baseline)			
	No (n = 1633; 93.4%)		Yes (n = 98; 6.6%)	
	n	%	n	%
Mania				
No	1602	98.2	92	93.8
Yes	31	1.8	6	6.2
Hypomania				
No	1616	98.9	97	99.3
Yes	17	1.1	1	0.7
Major depressive episode				
No	1334	82.7	76	78.5
Yes	299	17.3	22	21.5
Any mood disorder				
No	1311	81.1	72	74.5
Yes	322	18.9	26	25.5
Generalized anxiety disorder				
No	1575	96.6	92	93.4
Yes	58	3.4	6	6.6
Panic disorder				
No	1532	94.3	94	96.1
Yes	101	5.7	4	3.9
Any anxiety disorder				
No	1498	92.2	88	89.5
Yes	135	7.8	10	10.5
Alcohol audit score probable dependence or more				
No	1584	96.8	94	95.4
Yes	49	3.2	4	4.6
Drug abuse/dependence				
No	1573	96.0	94	95.1
Yes	60	4.0	4	4.9
Any substance use disorder				
No	1538	93.8	90	90.5
Yes	95	6.2	8	9.5

Notes: Prevalence of outcomes among those with and without IGD are presented as column percentages. Weighted %, unweighted frequencies.

**Table 2:** Baseline DSM-5 Internet Gaming Disorder as a predictor of individual and group of disorders at follow-up. Weighted crude and adjusted models.

	Crude Models			Adjusted Models		
	PR	95% CI	*p*-Value	aRR	95% CI	*p*-Value
Mania	3.51 *	(1.46, 8.42)	0.005	1.05	(1.00, 1.10)	0.075
Hypomania	0.64	(0.09, 4.83)	0.667	0.99	(0.97, 1.01)	0.300
Major depressive episode	1.24	(0.83, 1.84)	0.290	1.04	(0.96, 1.13)	0.283
Any mood disorder	1.35	(0.94, 1.93)	0.100	1.06	(0.98, 1.16)	0.163
Generalized anxiety disorder	1.95	(0.85, 4.48)	0.115	1.03	(0.98, 1.09)	0.250
Panic disorder	0.68	(0.24, 1.87)	0.452	0.98	(0.94, 1.02)	0.350
Any anxiety disorder	1.35	(0.72, 2.52)	0.348	1.02	(0.96, 1.09)	0.499
Alcohol audit score probable dependence or more	1.45	(0.52, 4.03)	0.480	1.01	(0.97, 1.05)	0.719
Drug abuse/dependence	1.21	(0.45, 3.27)	0.710	0.99	(0.94, 1.04)	0.683
Any substance use disorder	1.52	(0.76, 3.05)	0.241	1.01	(0.95, 1.08)	0.697

Notes. PR: Prevalence Ratio; aRR: adjusted Risk Ratio; CI: Confidence Interval. PRs and aRRs were estimated with generalized linear models (log link and binomial family) for each outcome and baseline DSM-5 IGD as the main exposure. Multivariable models were adjusted by sex, age, and mood, anxiety and substance use disorders at baseline. * *p* < 0.05.

## Data Availability

No data is publicly available for this project.
